# Effects of Catheter Tip Location on the Spread of Sensory Block Caused by a Continuous Thoracic Paravertebral Block: A Prospective, Randomized, Controlled, Double-Blind Study

**DOI:** 10.1155/2019/1051629

**Published:** 2019-05-14

**Authors:** Takayuki Yoshida, Yoshiko Watanabe, Takeshi Hashimoto, Atsushi Ohta, Tatsuo Nakamoto

**Affiliations:** ^1^Department of Anesthesiology, Kansai Medical University Hospital, Hirakata City, Osaka, Japan; ^2^Department of Anesthesiology, Niigata City General Hospital, Niigata City, Niigata, Japan; ^3^Department of Anesthesiology, Nagaoka Chuo General Hospital, Nagaoka City, Niigata, Japan; ^4^Department of Radiology and Radiation Oncology, Niigata University Graduate School of Medical and Dental Sciences, Niigata City, Niigata, Japan

## Abstract

Single injections in the anterior region of the thoracic paravertebral space (TPVS) have been reported to generate a multisegmental longitudinal spreading pattern more frequently than those in the posterior region of the TPVS. In this trial, we examined the hypothesis that a continuous thoracic paravertebral block (TPVB) administered through a catheter inserted into the anterior region of the TPVS allows a wider sensory block dispersion. Fifty consecutive patients undergoing video-assisted thoracic surgery were enrolled. Before the surgery, an infusion catheter was inserted into the TPVS through a needle placed adjacent to either the parietal pleura (group A) or internal intercostal membrane (group P) using an ultrasound-guided intercostal transverse approach according to a randomized allocation schedule. A chest radiograph was obtained postoperatively after injection of 10 mL of radiopaque dye through the catheter. Thereafter, 20 mL of 0.375% levobupivacaine was injected via the catheter, followed by commencement of continuous TPVB with 0.25% levobupivacaine at 8 mL/h. The primary outcome was the number of blocked dermatomes at 24 h after surgery. The secondary outcomes included radiopaque dye spreading patterns, the number of segments reached by the radiopaque dye, the number of blocked dermatomes at 2 h after surgery, and pain scores. The median (interquartile range [range]) number of blocked dermatomes 24 h after surgery was 3 (2.75–4 [1–6]) in group A (n = 22) and 2 (1.5–3 [0–7]) in group P (n = 25; p = 0.037). No significant differences in the other outcomes were found between the groups. In conclusion, a continuous TPVB administered using a catheter supposedly inserted into the anterior region of the TPVS allows a wider sensory block dispersion than a catheter inserted into the posterior region of the TPVS. This trial is registered with the UMIN Clinical Trials Registry (UMIN000018578).

## 1. Introduction

Thoracic paravertebral block (TPVB) achieved by injecting local anesthetic into the thoracic paravertebral space (TPVS) can provide unilateral somatic and sympathetic blockade at multisegmental levels [1–3]. However, factors affecting the distribution of a continuous TPVB have been rarely examined [4, 5]. The TPVS is continuous with the intercostal space laterally and is bounded by the parietal pleura anteriorly; the superior costotransverse ligament and internal intercostal membrane posteriorly; and the vertebrae, vertebral discs, and intervertebral foramina medially [1]. The TPVS contains the endothoracic fascia that has been reported to divide the TPVS into two compartments, i.e., an anterior extrapleural compartment and a posterior subendothoracic compartment [1, 6]. According to a previous study that investigated the spread of single-injection TPVB, injections of radiopaque dye supposedly made in the anterior extrapleural compartment of the TPVS tended to generate a multisegmental longitudinal spreading pattern when compared with that in the posterior subendothoracic compartment [7]. Moreover, according to an anatomic review article [8], each segment of the TPVS seems more seamlessly continuous with the cranially and caudally contiguous segments in the anterior part of the TPVS than in the posterior part. Therefore, we assume that local anesthetic continuously administered through a catheter inserted into the anterior part of the TPVS would reach more segments of the TPVS than that administered through a catheter inserted into the posterior part of the TPVS, thereby providing a wider spread of sensory block.

Among the various ultrasound-guided TPVB approaches [8], an intercostal transverse approach is superior in terms of allowing the operator to see the exact position of the needle tip because this technique is performed under in-plane ultrasound guidance using a high-frequency linear transducer [9]. This approach allows the needle tip position to be discriminated whether it is placed in the anterior or posterior part of the TPVS.

In this study, we tested the hypothesis that a continuous TPVB administered via a catheter threaded through a needle placed adjacent to the parietal pleura (i.e., the anterior part of the TPVS) using the intercostal transverse approach would provide a wider sensory block spread than that achieved via a catheter threaded through a needle placed adjacent to the internal intercostal membrane (i.e., the posterior part of the TPVS).

## 2. Materials and Methods

The study protocol was approved by the Research Ethics Committee of Niigata University School of Medicine (Niigata, Japan; identification no. 2245). The study was registered in the UMIN Clinical Trials Registry (http://www.umin.ac.jp/ctr/index.htm; identifier UMIN000018578) before the first participant was enrolled. The study was conducted at Niigata University Medical and Dental Hospital (Niigata, Japan). The patients were enrolled between August 2015 and March 2016 after they had provided written informed consent. The inclusion criteria were American Society of Anesthesiologists physical status 1–3 and scheduling for unilateral video-assisted thoracic surgery. The following exclusion criteria were applied: inability to communicate lucidly; age <20 years or >80 years; body mass index >30; weight <40 kg; allergy or contraindications to any of the drugs used in the study; preexisting sensory disturbance on the trunk; prior thoracotomy on the ipsilateral side; renal or hepatic failure; infection at the injection site; placement of a posterolateral thoracotomy; and intrathoracic catheter misplacement.

Fifty consecutive patients were randomly assigned to receive a continuous TPVB through a catheter inserted into either the anterior part (group A) or the posterior part (group P) of the TPVS. The randomization was established using a computer-generated randomization sequence (http://www.randomization.com), in blocks of 10, and concealed using sealed prenumbered opaque envelopes prepared by a research assistant not involved in the study. An anesthesiologist performing the catheterization for the continuous TPVB opened the envelope just after completion of induction of general anesthesia.

None of the patients received premedication. Bispectral index and standard monitoring were performed in all enrolled patients. General anesthesia was induced using a target-controlled infusion of propofol (target blood concentration 2.0–6.0 *μ*g/mL) and a continuous infusion of remifentanil (0.25–0.5 *μ*g/kg/min). Subsequently, intravenous rocuronium 0.9 mg/kg was administered to facilitate tracheal intubation. Anesthesia was maintained with a target-controlled infusion of propofol, a continuous infusion of remifentanil, and intermittent boluses of rocuronium at the discretion of the anesthesiologist who managed the general anesthesia during surgery. The bispectral index was maintained between 40 and 60.

**Figure 1:**
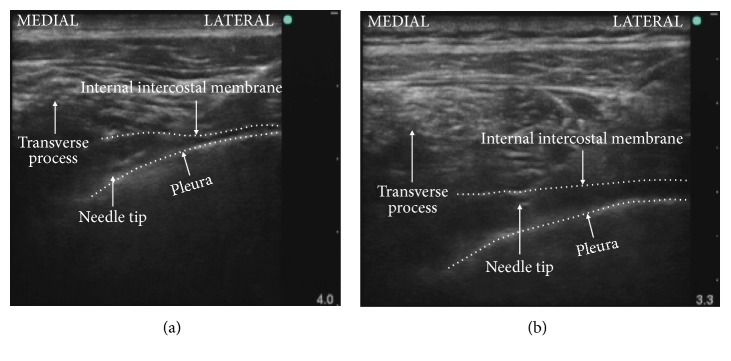
The final position of the Tuohy needle tip on an ultrasound image defining group A (a) and group P (b).

Three anesthesiologists (TY, TH, and YW), who each had performed more than 50 catheterization procedures for continuous TPVB using the ultrasound-guided intercostal transverse approach, placed all the TPVS catheters. Immediately after induction of general anesthesia, the patient was placed in the lateral decubitus position with the side to be blocked uppermost. After ensuring skin asepsis, a 6–13-MHz linear transducer (HFL 38x; FUJIFILM SonoSite Inc., Tokyo, Japan) inside a sterile sheath and connected to an ultrasound apparatus (S-Nerve; FUJIFILM SonoSite Inc.) was placed on the fifth intercostal space to see a transverse image of the lateral apex of the TPVS, transverse process, internal intercostal membrane, and pleura as described previously [9]. If these structures could not be seen clearly through the fifth intercostal space, the transducer was moved to either the sixth or fourth intercostal space in order to obtain the appropriate image. Next, an 18-gauge Tuohy needle (Perican II; B. Braun AG, Melsungen, Germany) was inserted and advanced in plane with the transducer, with the bevel of the needle tip facing posteriorly in a lateral-to-medial direction toward the TPVS. At 0.5–1.5 cm lateral to the most posterior border of the transverse process, the needle tip was placed immediately anterior to the internal intercostal membrane in group P and immediately posterior to the parietal pleura in group A (Figure 1). After confirmation of the correct needle tip position using ultrasound in accordance with the group allocation, 8 mL of normal saline was injected through the needle, and ventral movement of the pleura was confirmed by ultrasound. Subsequently, the bevel of the needle tip was rotated 180 degrees to face anteriorly; next, a 20-gauge catheter (Perifix SoftTip Catheter; B. Braun AG) was threaded 4.5 cm beyond the needle tip in a medial direction. The catheter was fixed to the skin with a suture. Approximately 20 min before the beginning of surgery, 20 mL of 0.375% levobupivacaine was injected through the catheter. No additional local anesthetic was injected during surgery.

After completion of surgery, the patient's position was changed to supine, and a radiography film cassette was placed below the back of the thorax. A chest radiograph was taken following injection of 10 mL of radiopaque dye (Omnipaque 240; Daiichi-Sankyo Pharmaceutical, Tokyo, Japan) through the TPVS catheter over approximately 30 sec. Twenty milliliters of 0.375% levobupivacaine was then injected through the catheter, followed by commencement of a continuous thoracic paravertebral infusion of 0.25% levobupivacaine at 8 mL/h using an elastomeric disposable pump (Vessel Fusor; AuBEX, Tokyo, Japan).

After emergence from general anesthesia, intravenous sugammadex 4 mg/kg was administered to reverse neuromuscular blockade, after which the patient's trachea was extubated.

### 2.1. Study Parameters and Statistical Analysis

**Figure 2:**
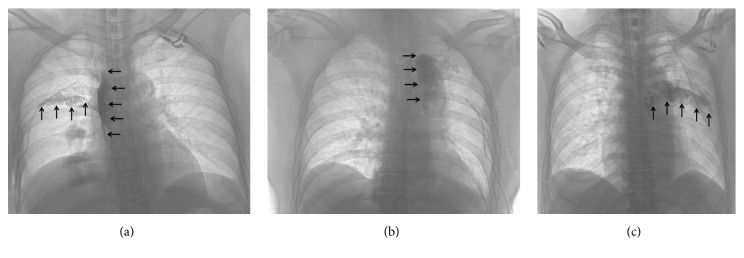
Spreading pattern of radiopaque dye injected using a thoracic paravertebral block technique. (a) The spread in the thoracic paravertebral space shown along the lateral side of the thoracic vertebrae and the intercostal spread. (b) The cloud-like spread. (c) The intercostal spread alone. The black arrows indicate the spread of the radiopaque dye.

The primary outcome of the study was the number of dermatomes that were blocked 24 h after surgery. The secondary outcomes were the radiopaque dye spreading patterns, the number of segments reached by the radiopaque dye, the number of dermatomes that were blocked 2 h after surgery, and pain scores at rest, during coughing, and during movement 2 and 24 h after surgery. The blocked dermatomes were evaluated using an ice pack in a standardized manner. The blocked area was tested on the midclavicular line from the T4 dermatome on the surgical side. The dermatomes at which the patient perceived less or no sensation to the cold stimulus when compared with those on the contralateral side were registered as blocked dermatomes. If less sensation to the cold stimulus was suspected on the contralateral side (i.e., the nonsurgical side), dermatomes at which the patient perceived less or no sensation to the cold stimulus when compared with those on the neck were registered as the blocked dermatomes. The number of blocked dermatomes was recorded on a dedicated dermatome map. Pain was assessed using a numerical rating scale (0, no pain; 10, worst pain imaginable). These outcomes were investigated by anesthesiologists who were blinded to the group allocation. A previous report by Naja et al. [7], which investigated the distribution of radiopaque dye administered using a single-injection TPVB technique, classified radiopaque dye spreading patterns on a chest radiograph into the following four types: TPVS spread, cloud-like spread, intercostal spread, and combinations of these three types of spread (Figure 2). A radiologist (AO) blinded to the group allocation determined the spreading pattern of the radiopaque dye in the present study using the same classification method. The presence of radiopaque dye in the prevertebral or epidural space was also checked [10, 11]. The radiologist also evaluated the number of segments reached by the dye. The TPVS or intercostal space where the radiopaque dye was observed on the chest radiograph, even in a small amount, was registered as the segment reached by the dye.

Sample size calculations were based on the hypothesis that a continuous TPVB administered through a catheter inserted into the anterior part of the TPVS using the intercostal transverse approach would allow a wider spread of sensory block than that administered through a catheter inserted into the posterior part of the TPVS. To this end, the primary outcome was the number of dermatomes that were blocked 24 h after surgery. The prespecified analysis of the primary outcome was performed using the Mann-Whitney* U* test. However, the original power analysis was based on an approximation using a two-tailed Student's* t*-test. According to a pilot study (n = 4), the mean (standard deviation) number of dermatomes that were blocked 24 h after surgery in patients who received a continuous thoracic paravertebral infusion via a catheter inserted through a Tuohy needle that was placed adjacent to the parietal pleura, was 3.75 (0.96). We considered that achieving one additional blocked dermatome by providing a continuous TPVB through a catheter placed at the anterior part of the TPVS was clinically relevant. With a statistical power of 0.8 and a type I error rate of 0.05, the sample size calculation indicated that a minimum of 16 patients per group was needed to detect this difference. Twenty-five patients per group were enrolled in the study to allow for dropouts. Sample sizes were calculated using G*∗*Power software version 3.1.9.2 for Mac OS X (University of Heinrich-Heine, Dusseldorf, Germany).

To analyze differences between the two groups, a two-tailed Student's* t*-test was applied for normally distributed continuous data. The Mann-Whitney* U* test was used to analyze non-normally distributed continuous data and noncontinuous data. Normality of distribution was assessed using the Shapiro-Wilk test. Categorical data were compared using Fisher's exact test. A p-value of < 0.05 was considered statistically significant. No adjustments were made for multiple comparisons in this study. Hence, significant findings in secondary outcomes should be interpreted as suggestive, requiring confirmation in a future study. The statistical analyses were conducted using GraphPad Prism 7 for Mac OS X version 7.0e (GraphPad Software, San Diego, CA, USA).

## 3. Results

**Figure 3:**
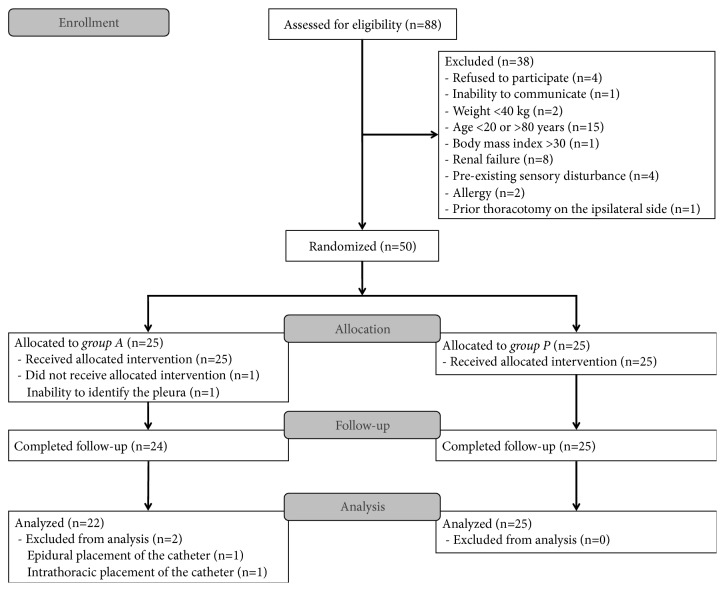
CONSORT flow diagram.

Fifty consecutive patients were randomized. However, one patient assigned to group A did not receive his allocated intervention because of poor visibility of the parietal pleura under ultrasound guidance. All the other patients received their allocated interventions. No patients were lost to follow-up; however, two patients in group A were excluded from the final analysis. In one patient, a chest radiograph following injection of the radiopaque dye revealed epidural placement of the catheter. Intrathoracic placement of the catheter was found intraoperatively in the other patient. Finally, 22 patients in group A and 25 patients in group P were included in the analysis (Figure 3). The baseline and perioperative characteristics of the study patients are shown in Table 1. The paravertebral catheters were inserted through the fifth intercostal space in all patients except for one in group P in whom the catheter was introduced through the sixth intercostal space. The mean (standard deviation) length of the needle insertion was 4.71 (0.48) cm in group A and 4.57 (0.50) cm in group P (P = 0.36).

**Figure 4:**
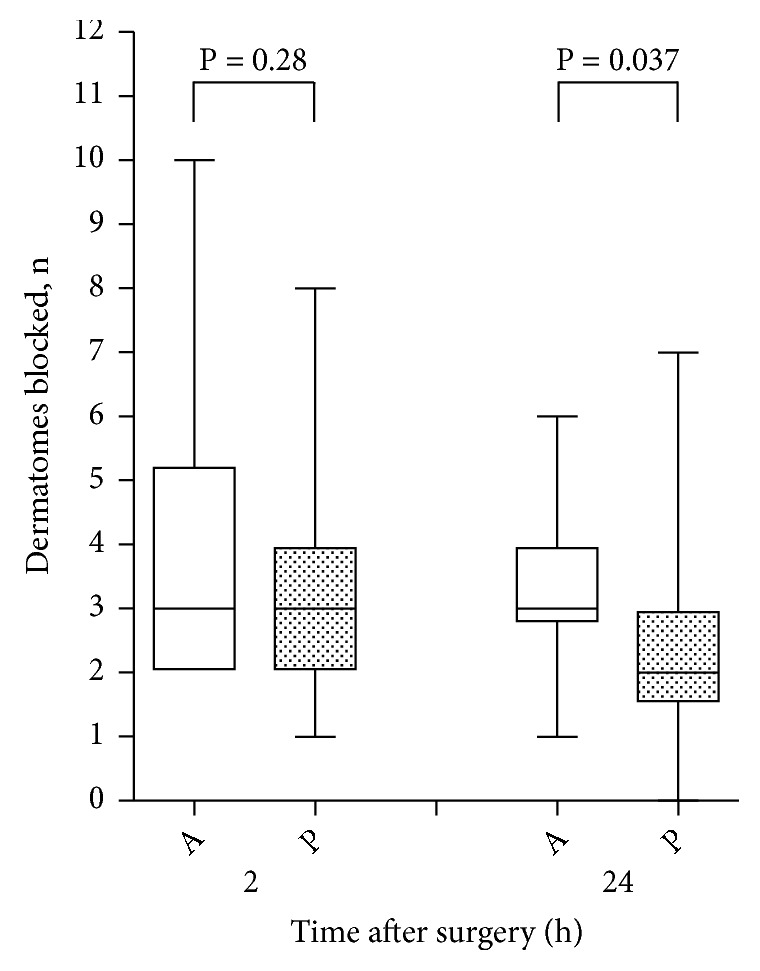
Number of dermatomes blocked at 2 and 24 h after surgery in patients receiving a continuous thoracic paravertebral block. A and P indicate group A and group P, respectively. The horizontal lines indicate the medians, the boxes indicate the interquartile ranges, and the whiskers indicate the ranges. The P-values were calculated using the Mann-Whitney* U* test.

Figure 4 shows the number of dermatomes that were blocked 2 and 24 h after surgery. For the primary endpoint of this study, the median (interquartile range [range]) number of blocked dermatomes at 24 h after surgery was determined to be 3 (2.75–4 [1–6]) in group A and 2 (1.5–3 [0–7]) in group P (95% confidence interval of difference in medians, -2 to 0; P = 0.037). The numbers of segments reached by the radiopaque dye and the dye spreading patterns were not significantly different between the groups (Table 2). One patient in each group showed prevertebral spread of the radiopaque dye on a chest radiograph. Contralateral spread of sensory block was not observed in any of the study patients, except in one patient in group P in whom the catheter tip was found to be in the epidural space based on the spread of the dye on a chest radiograph. Postoperative pain scores did not differ significantly between the groups (Table 3).

## 4. Discussion

This randomized, double-blind, controlled trial demonstrated that a continuous TPVB administered through a catheter inserted adjacent to the parietal pleura provided wider spread of sensory block at approximately 24 h after commencement of the continuous infusion than a block administered through a catheter inserted adjacent to the internal intercostal membrane. However, the dye spreading patterns and the number of segments reached by the dye were similar regardless of the catheter position.

According to a previous anatomic study that illustrated various ultrasound-guided TPVB approaches [8], each segment of the TPVS seems to connect with both the cranial and caudal contiguous segments at the anterior part of the TPVS. Another anatomic report published after the present study was completed also supports this finding [12]. Taketa et al. compared the analgesic effects of continuous TPVB produced using two different ultrasound-guided approaches and demonstrated that a paralaminar approach provided sensory block that covered a wider range than that covered by the intercostal transverse approach [13]. Using the paralaminar approach, a needle tip penetrated the skin perpendicularly and reached inside the TPVS. Next, a catheter was inserted in a purely anterior direction. However, with the intercostal transverse approach, a catheter was threaded into the TPVS from a needle tip placed immediately lateral to the TPVS. Therefore, the tip of a catheter placed using the paralaminar approach would theoretically be situated at the more anterior part of the TPVS than a catheter placed using the intercostal transverse approach. Thus, the results reported by Taketa et al. are consistent with our hypothesis and the findings of our present study. We believe that the main factor contributing to our results was the longitudinal connection of the contiguous segments at the anterior part of the TPVS.

The impact of the endothoracic fascia on the spread of a TPVB is controversial. Some papers have reported that the endothoracic fascia separates the TPVS into ventral and dorsal compartments [1, 6] while another clinical study demonstrated that injection of radiopaque dye supposedly ventral to the endothoracic fascia allowed a wider spread of dye longitudinally than that dorsal to the endothoracic fascia [7]. However, a recent anatomic evaluation found that the endothoracic fascia was indistinguishable from the parietal pleura in human cadavers, and internal subdivision of the TPVS by the endothoracic fascia was not observed [12]. Moreover, Fujii et al. reported that local anesthetic injected into the TPVS can communicate with the interpleural space by permeating the parietal pleura, probably working as an interpleural block [14]. This communication through the parietal pleura may explain the previously reported discrepancy in distribution between radiopaque dye or local anesthetic spreading patterns, which were assessed by imaging modalities, and the actual spread of sensory block [15, 16]. We suspect that local anesthetic administered into the anterior part of the TPVS, which is adjacent to the parietal pleura, would be more likely to permeate through the parietal pleura into the interpleural space than local anesthetic administered into the posterior part, partially contributing to the wider distribution.

Contrary to a previous report [7], there was no difference in the radiopaque dye spreading patterns or the number of segments reached by the dye between the groups. In the previous study, the radiopaque dye was injected directly into the TPVS through a needle whereas the dye was injected via the indwelling catheter in our study. This inconsistency may reflect the limited injection speed of the radiopaque dye because of its high viscosity as well as the narrow catheter. Furthermore, we did not confirm the position of the catheter tip, although the position of the needle tip was determined under ultrasound guidance. The catheter tip might be threaded toward an unintended direction in some patients [17]. Indeed, prevertebral spread of the radiopaque dye, which would be more likely to occur via the anteriorly inserted catheter, was observed even in group P.

Some recent papers have demonstrated that programmed intermittent administration of a bolus is superior to a continuous infusion in terms of producing a wider spread of sensory block by a TPVB [18, 19]. Furthermore, the distribution of a single bolus injection of TPVB was reported to reach more longitudinally when the solution was injected into the anterior part of the TPVS [7], as mentioned in the Introduction. Therefore, the difference in spread of sensory blockade according to location of the catheter tip might be greater with an intermittent bolus injection than with a continuous infusion. However, this assumption needs to be validated in a future study.

This study has several other limitations. First, the wide heterogeneity in terms of surgical nociception because of differences in the surgical procedure performed might have prevented correct evaluation of the difference in pain scores between the groups. However, our first priority in this study was to clarify the spread of sensory block according to the location of the catheter. Second, the appropriate length of the catheter beyond the needle tip was not examined. We set the length of the catheter beyond the Tuohy needle tip at 4.5 cm uniformly in this study; however, unintended epidural and prevertebral placements of the catheter occurred in some patients. A shorter catheter insertion length might have been desirable to ensure differentiation of the catheter position more precisely, although there is presently no definite recommendation regarding the catheter insertion length for a continuous TPVB. Finally, we did not assess the risk or feasibility of placing a needle very close to either the parietal pleura or the internal intercostal membrane. Placing the needle tip very close to the pleura should be avoided in certain patients, for example, those in whom visibility of the parietal pleura is poor. Indeed, interpleural placement of the catheter occurred in one patient in group A. A potential drawback of positioning the needle tip immediately anterior to the internal intercostal membrane is that it might be unintentionally moved to outside the intercostal space before insertion of the catheter. Two patients in group P showed no sensory blockade 24 h after surgery, and ultrasound guidance should have been used to observe the catheter being threaded into the TPVS through the needle.

## 5. Conclusions

A continuous TPVB administered through a catheter inserted adjacent to the parietal pleura using the intercostal transverse approach allows a wider spread of sensory block than that administered via a catheter inserted adjacent to the internal intercostal membrane. Insofar as visibility of both the parietal pleura and the needle tip under ultrasound guidance can be guaranteed, we recommend placing the needle tip close to the parietal pleura before threading a catheter through the needle into the TPVS in order to achieve a wider spread of sensory block.

## Figures and Tables

**Table 1:** Baseline and perioperative characteristics of patients in the study.

	Group A (n = 22)	Group P (n = 25)
Age, years	68 (8)	65 (12)
Sex, female	10 (45)	8 (32)
Height, cm	160.8 (9.7)	162.1 (7.8)
Weight, kg	57.2 (9.1)	57.3 (10.0)
Body mass index	22.1 (2.3)	21.7 (3.0)
ASA physical status, 1/2/3	1/19/2	1/21/3
Surgical side, right	12 (55)	15 (60)
Diagnosis, primary lung cancer/metastatic lung cancer/pneumothorax/ interstitial pneumonitis	15/3/1/3	16/4/3/2
Procedure, lobectomy/segmentectomy/wedge resection	12/5/5	10/5/10
Operating time, min	162 (65)	141 (70)
Anesthesia time, min	256 (65)	237 (77)

The data are shown as the mean (standard deviation) or as the number (proportion).

ASA: American Society of Anesthesiologists.

**Table 2:** Distribution of radiopaque dye.

	Group A (n = 22)	Group P (n = 25)	P-value
Number of segments reached by the dye	3 (2.75–4 [2–5])	3 (2–4 [1–6])	0.81
Types of spread			
TPVS + IC	11	11	0.10
TPVS + IC + PREV	1	1	
TPVS + CL	1	0	
CL	6	2	
CL + IC	0	4	
IC	3	7	

The data are shown as the median (interquartile range [range]) or number. The P-values were calculated using the Mann-Whitney *U* test or Fisher's exact test as appropriate.

TPVS: spread in the thoracic paravertebral space; IC: intercostal spread; PREV: prevertebral spread; CL: cloud-like spread.

**Table 3:** Postoperative pain scores on the numerical rating scale.

	Group A (n = 22)	Group P (n = 25)	P-value
Pain at rest			
2 h after surgery	2.5 (0.75–4 [0–6])	2 (0–3 [0–6])	0.32
24 h after surgery	1 (0–2 [0–4])	2 (0–3 [0–5])	0.20
Pain during coughing			
2 h after surgery	3 (1–5 [0–7])	2 (0–4 [0–7])	0.44
24 h after surgery	2.5 (1.75–4 [0–7])	3 (2–5 [0–7])	0.15
Pain during movement			
2 h after surgery	3 (1–4.25 [0–8])	2 (0.5–4.5 [0–8])	0.51
24 h after surgery	2.5 (1.75–4.25 [0–6])	4 (2.5–5 [0–7])	0.075

The data are shown as the median (interquartile range [range]). The P-values were calculated using the Mann-Whitney *U* test.

## Data Availability

The raw clinical data used to support the findings of this study are available from the corresponding author upon reasonable request.
